# Supplementary material for: A streptococcal lipid toxin induces membrane permeabilization and pyroptosis leading to fetal injury
Source: EMBO Mol Med. 2015 Mar 6;7(4):488–505. doi: 10.15252/emmm.201404883 (PMC4403049; doi:10.15252/emmm.201404883)
Supplement: Supplementary file 1 — Supplementary Figures S1–S9 [file emmm0007-0488-sd1.pdf]

SUPPLEMENTARY INFORMATION

TABLE OF CONTENTS

1) Figure S1. Kinetics of K^+ and Hb efflux due to hemolysis induced by *Staphylococcus aureus* α-toxin and Triton X 100.
2) Figure S2. Osmoprotectants do not protect from direct lysis.
3) Figure S3. Disruption of artificial lipid bilayers by GBS pigment (75nM), pore forming porin MspA of *Mycobacterium smegmatis* and detergent SDS.
4) Figure S4. The GBS pigment induces secretion of IL18 but not IL6, TNF- α and IFN-γ from THP-1 derived macrophages.
5) Figure S5. Western blots of THP-1 shRNA knockdown cell lines.
6) Figure S6. Osmoprotectants and the caspase 3/7 inhibitor do not provide protection from macrophage cell death observed with hyperpigmented GBS strains.
7) Figure S7. Increasing amounts of the caspase inhibitor z-YVAD-FMK provided significant protection from GBS pigment mediated cell death in macrophages.
8) Figure S8. Nucleic acid or proteins are absent from purified GBS pigment.
9) Figure S9. Inactive GBS pigment is not hemolytic and does not induce cell death or IL-1β secretion in THP-1 macrophages.
10) Reference Cited

Figure S1. Kinetics of K^+ and Hb efflux due to hemolysis induced by *Staphylococcus aureus* α-toxin and Triton X 100.

(A) Release of K^+ and Hb release was measured from RBC treated with 0.47µM *S. aureus* α-toxin. Efflux of K^+ occurred faster than Hb, as measured by time to 50% release (9.6min vs 13.3min, $p < 0.001$, extra sum-of-squares F test); this lag is similar to the lag observed for GBS pigment-mediated hemolysis (see Fig. 1B). Data are the average ± SEM of two independent experiments. (B) K^+ and Hb release was measured from RBC treated with 0.22µM Triton-X 100 and 100% release of K^+ and Hb occurred instantly.

A

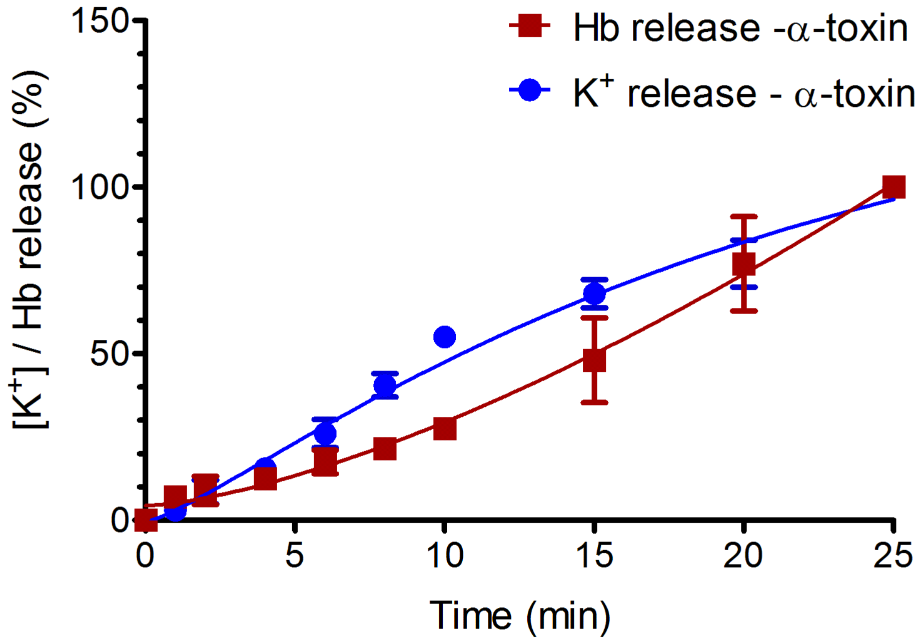

B

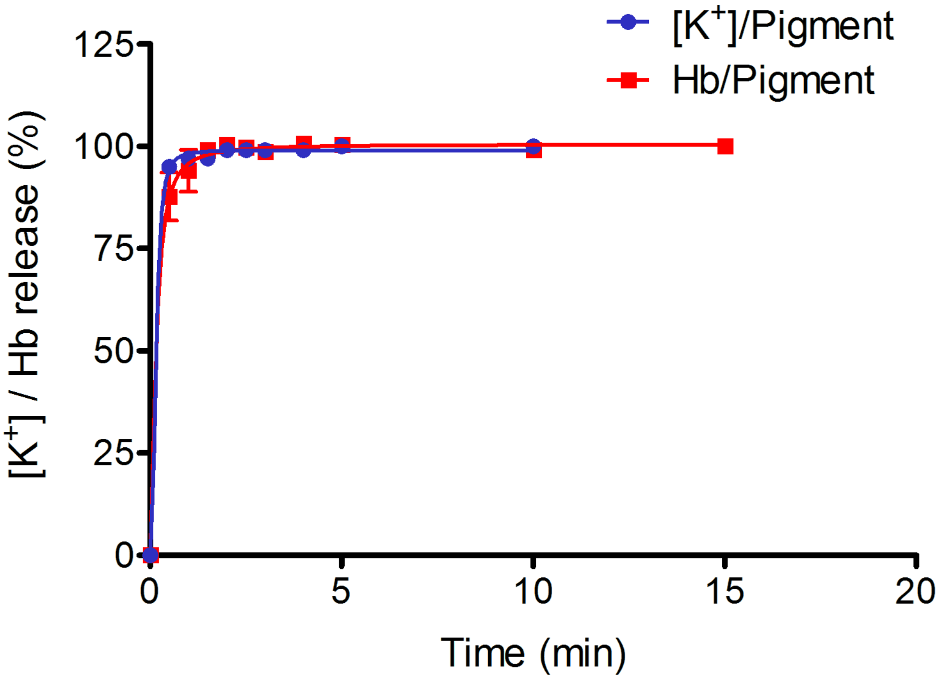

Figure S2. Osmoprotectants do not protect from direct lysis.

Osmoprotectants provide minimal protection from hemolysis caused by SDS (a direct lysis mechanism). Notably, the osmoprotectants PEG1500 and PEG3000 provided complete protection to lysis induced by the GBS pigment, suggesting that the pigment induces hemolysis not via a direct lysis mechanism, but rather a colloidal-osmotic mechanism (see Fig. 1C). Data are the average ± SEM of two independent experiments.

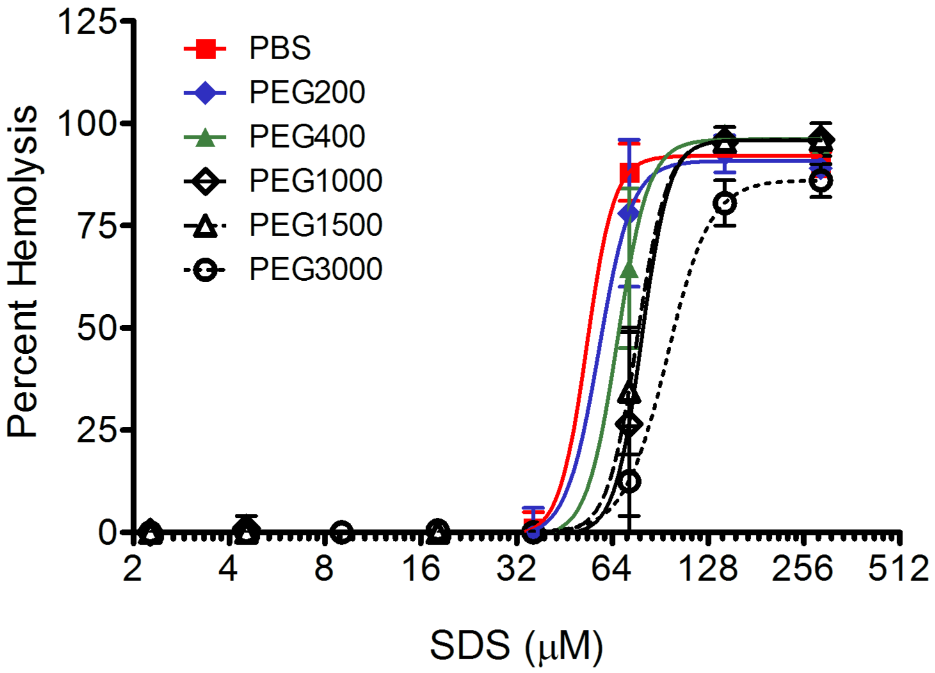

Figure S3. Disruption of artificial lipid bilayers by GBS pigment (75nM), pore forming porin MspA of *Mycobacterium smegmatis* and detergent SDS. (A, B) GBS pigment (75nM) induces membrane permeability in black lipid membranes (BLMs), see A & for detail *t* = 320-345s, see B. Equivalent volume of Δ*cylE* extract does not disrupt BLMs (see C). As controls, BLMs were incubated with MspA (0.51nM; D) or SDS (350µM; E). For MspA, protein was added at *t* = 0s, and mixed by pipetting from *t* =10-20s. Stepwise increase in current indicative of multiple pore formation is observed. For SDS, detergent was added at *t*=0s and mixed by pipetting from *t* =17-22s. A rapid and large increase in conductance is observed around 23s which is sustained for a few seconds before bilayer disruption, indicative of bilayer solubilization.

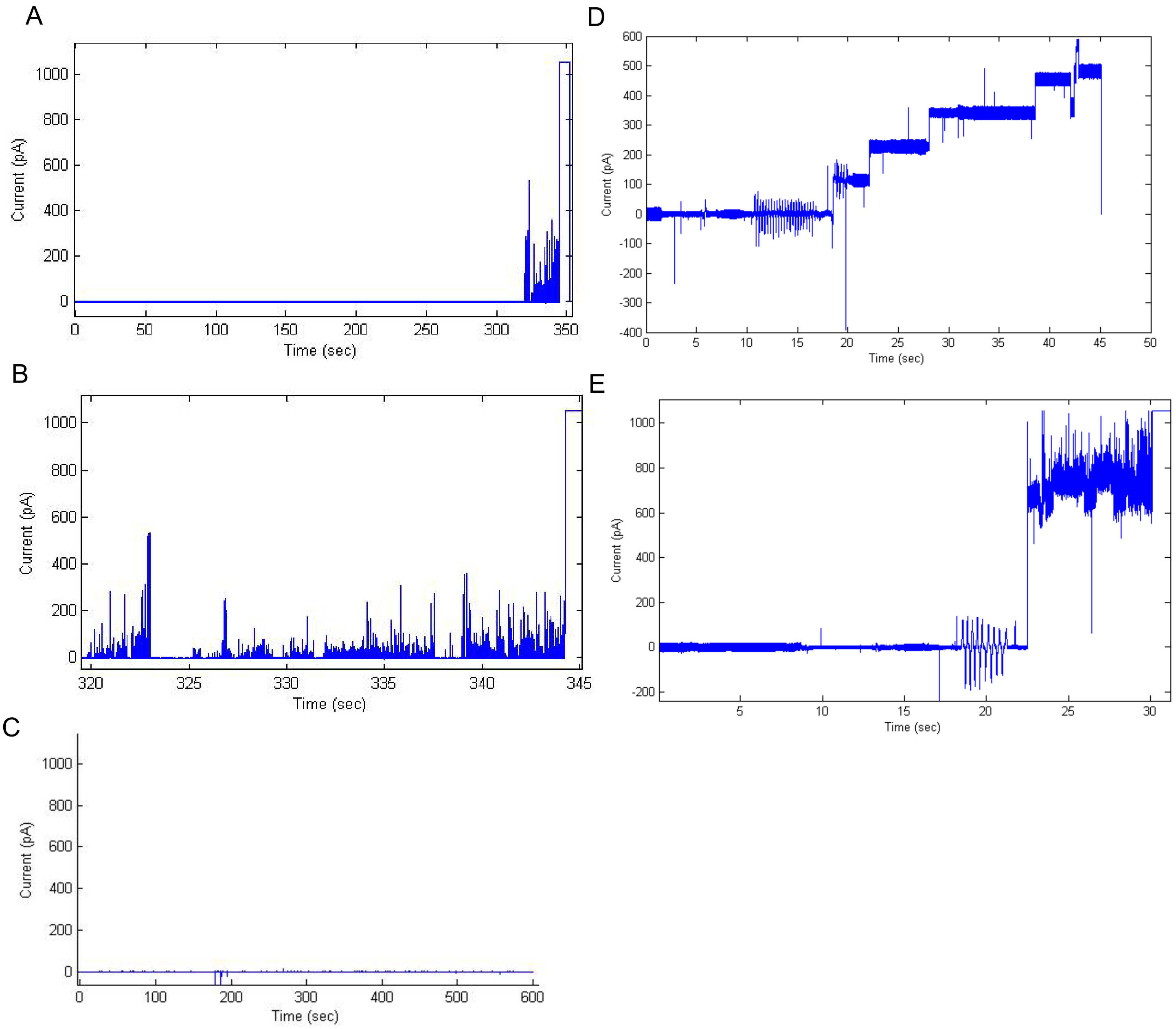

Figure S4. The GBS pigment induces secretion of IL18 but not IL6, TNF- α and IFN-γ from THP-1 derived macrophages.

Levels of IL-6, TNF-α, and IFN-γ or IL-18 in the supernatant of pigment or Δ*cylE* extract treated THP-1 cells were measured. While secretion of IL-18 is significantly higher in pigment treated cells, there is no significant increase in IL-6, TNF-α, or IFN- in response to purified GBS pigment. This suggests that the purified pigment induces activation of the NLRP3 inflammasome, it does not induce secretion of TLR-mediated cytokines ($n=3$, ***p = 0.0002, *p = 0.017 for 0.5 μM pigment, *p = 0.025; for 0.25μM pigment, Bonferroni's multiple comparison test following ANOVA; error bars ± SEM).

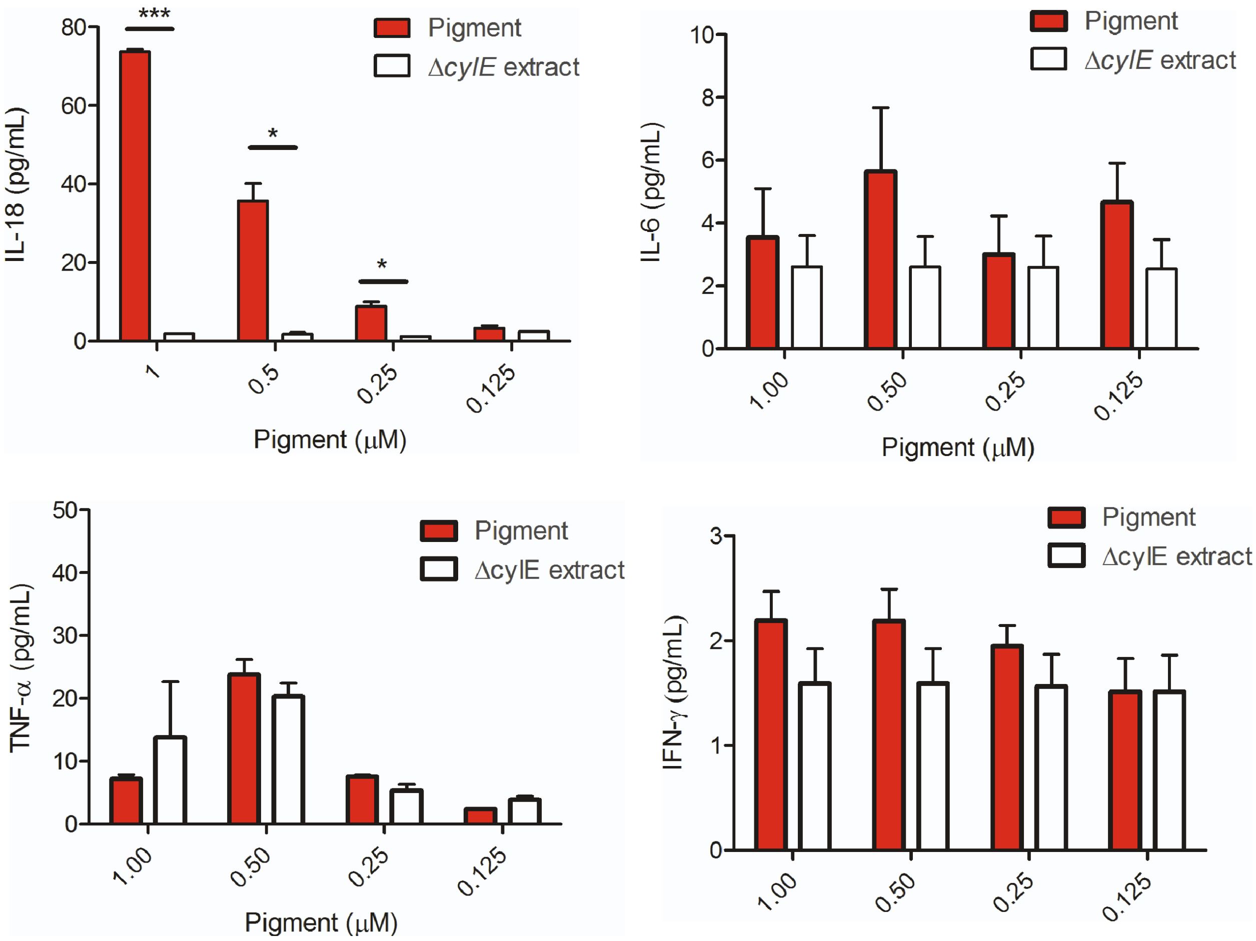

Figure S5. Western blots of THP-1 ShRNA knockdown cell lines.

Western blots demonstrate the lack of ASC and NLRP3 in the shRNA knockdown THP-1/shASC, and THP-1/shNLRP3 cell lines, respectively. Briefly, equal amounts of whole cell lysates from $5x10^6$ cells of the PMA-differentiated THP-1 cell lines (THP-1/vector, THP-1/scrambled, THP-1/shASC, THP-1/shNLRP3) were resolved on SDS-PAGE and probed by western blotting for either ASC or NLRP3 using anti-ASC (1μg/ml; Enzo Life Science) and anti-NLRP3/NALP3 antibodies (1μg/ml; Adipogen), respectively. For comparison, equal amounts of protein on SDS-PAGE were also probed for actin using an anti-actin antibody (0.05μg/mL; GenScript). The results below indicate that unlike the THP-1/vector and THP-1/scrambled cell lines, signal corresponding to ASC and NLRP3 is significantly reduced if not completely absent in the THP-1/shASC cell line (lane 2) and THP-1/shNLRP3 cell line (lane 4), respectively.

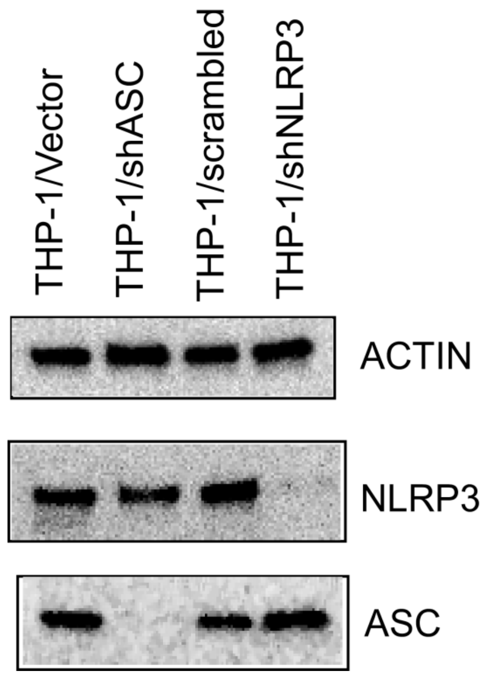

Figure S6. Osmoprotectants and the caspase 3/7 inhibitor do not provide protection from macrophage cell death observed with hyperpigmented GBS. THP-1 cells transfected with empty vector, scrambled control, shASC or shNLRP3 were incubated with GBSΔ*covR* (MOI=1) for 4 hours in the presence or absence of 30mM PEG1500 (A) or 100μM caspase 3/7 inhibitor (Z-DEVD-FMK, shown below as DEVD or with control DMSO (B). Percent cell death was then measured by LDH release. The addition of the osmoprotectant (PEG1500) or DEVD did not reduce the amount of cell death in THP-1 derived macrophages including shASC and shNLRP3 cells.

A

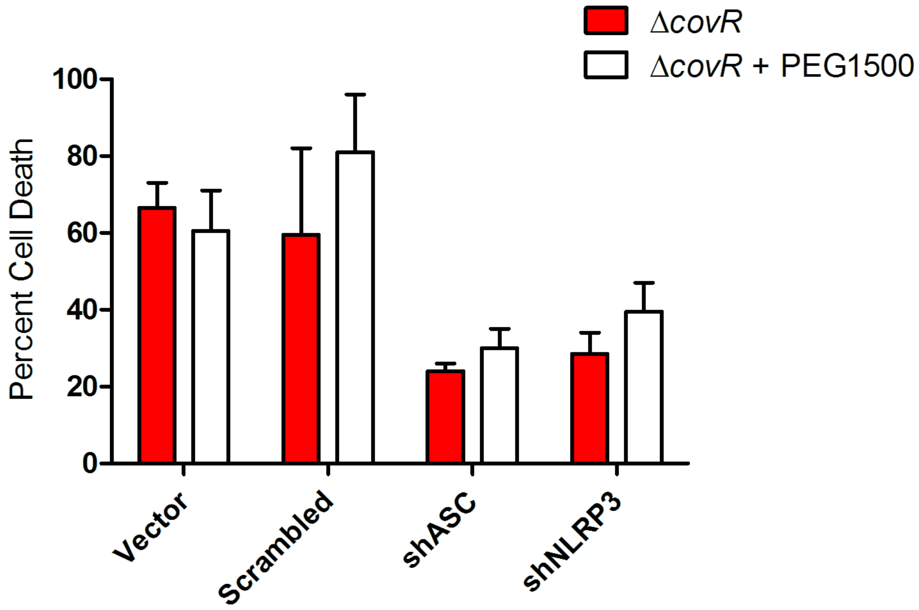

B

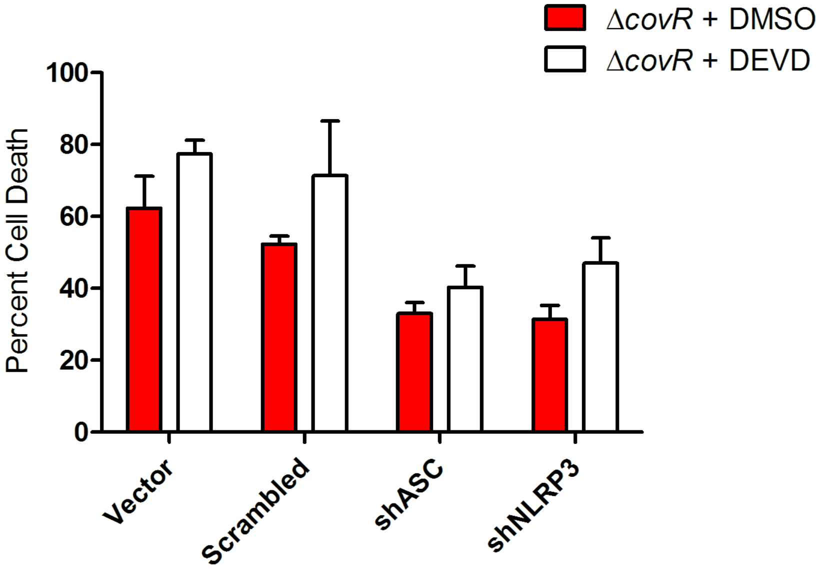

Figure S7. Increasing amounts of the caspase I inhibitor z-YVAD-FMK provided significant protection from GBS pigment mediated cell death in macrophages. WT THP-1 macrophages were treated with increasing amounts of the the caspase 1 inhibitor Z-YVAD-FMK or control 'DMSO only' prior to treatment with the GBS pigment. At 200μM Z-YVAD-FMK, significant if not complete protection from GBS pigment mediated cell death was observed. Data are average of two independent experiments with independent batches of purified pigment used in both experiments; error bars ± SEM. Bonferroni's multiple comparison test following ANOVA, (*n=2,* *** *p* = 0.0001, **p* = 0.044 for 4μM pigment, 100 vs. 200 μM YVAD; **p* = 0.049 for 2μM pigment, DMSO only vs. 200 μM YVAD; * *p* = 0.023 for 2μM pigment, 50 vs. 200 μM YVAD).

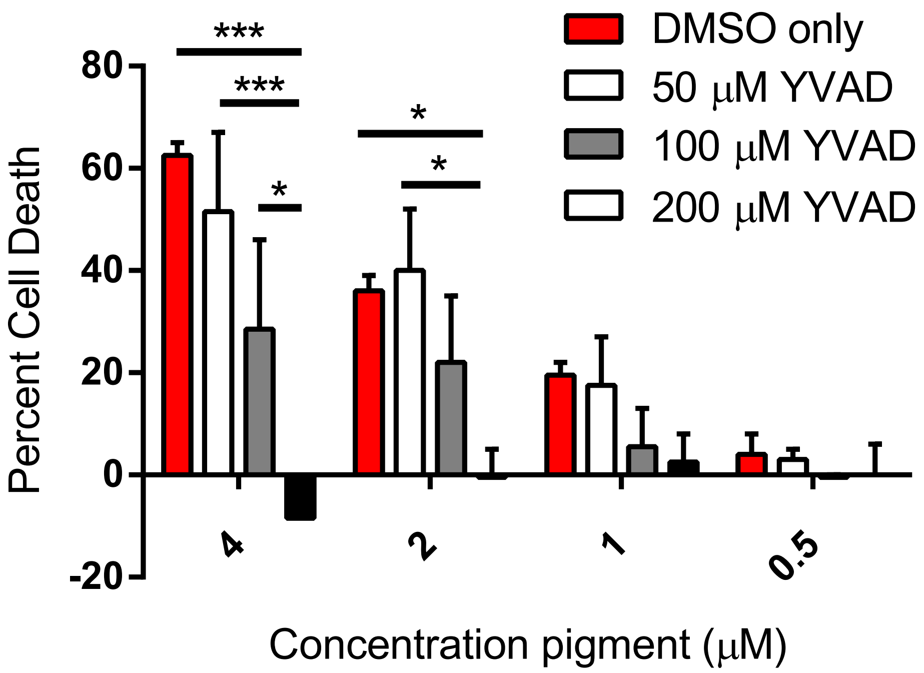

Figure S8. Nucleic acid or proteins are absent from purified GBS pigment.

(Left Panel) Purified GBS pigment (1.6nmol) and equivalent amount of control Δ*cylE* extract were resolved by agarose gel electrophoresis and stained with ethidium bromide for detection of nucleic acids (DNA or RNA).
(Middle Panel) Purified GBS pigment (9.3nmol) and equivalent amount of control Δ*cylE* extract were resolved by SDS-PAGE followed by Sypro Ruby staining for detection of proteins.
(Right Panel) Three independently purified batches of GBS pigment (0.6nmol in 3μL) and Δ*cylE* extract were tested for the presence of GBS RNA using RT-PCR (Qiagen); reverse transcription and PCR for the housekeeping gene *rpsL* was performed as previously described (Lembo et al, 2010). As a control, 500ng of RNA isolated from the GBS strain A909 was tested in the presence and absence of DMSO:0.1% TFA.

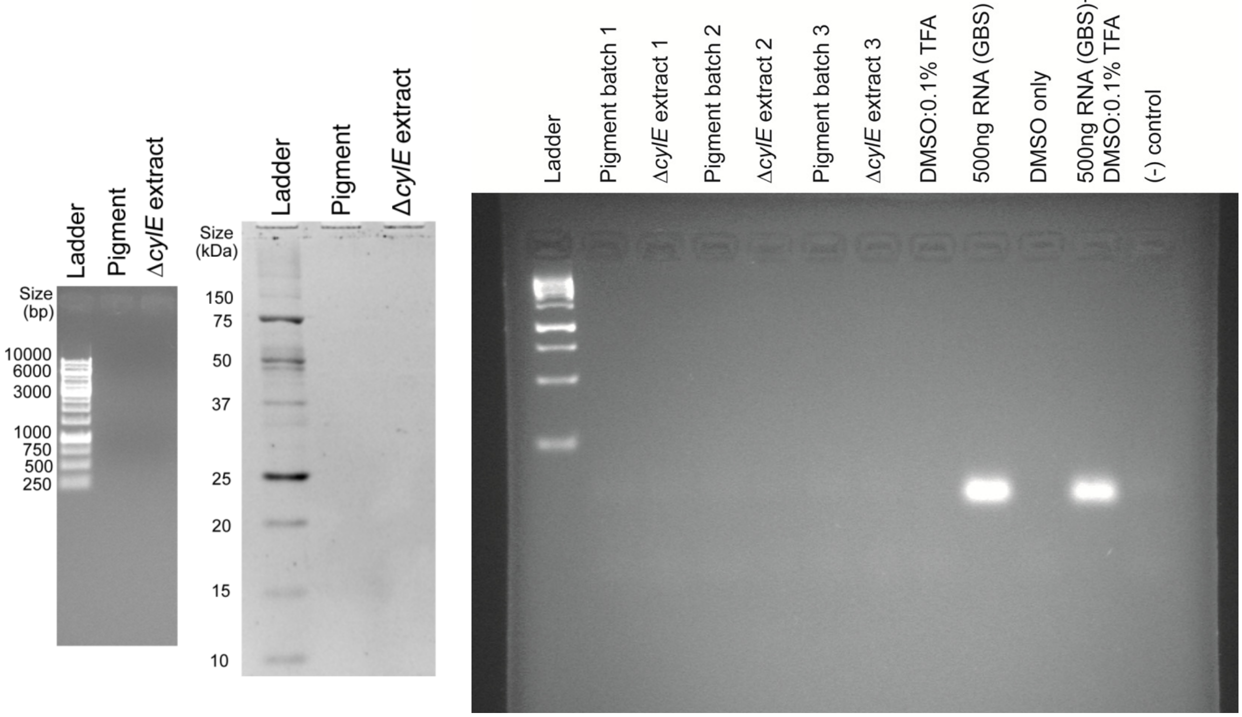

Figure S9. Inactive GBS pigment is not hemolytic and does not induce cell death or IL-1β secretion in THP-1 macrophages.

(A) GBS pigment dissolved in DMSO:0.1%TFA:20% starch is hemolytic whereas GBS pigment dissolved only in DMSO:0.1%TFA (without starch) is not hemolytic.

(B, C) Inactive, nonhemolytic GBS pigment (due to the absence of starch) does not induce cell death (see B) or IL1β release (see C) in THP-1 macrophages. Data are average of three independent experiments, error bars ± SEM (n=3, **** p = 0.0002, *** p = 0.001, *p = 0.035 for 1μM pigment, *p = 0.0097 for 0.5 μM pigment; Bonferroni's multiple comparison test following ANOVA).

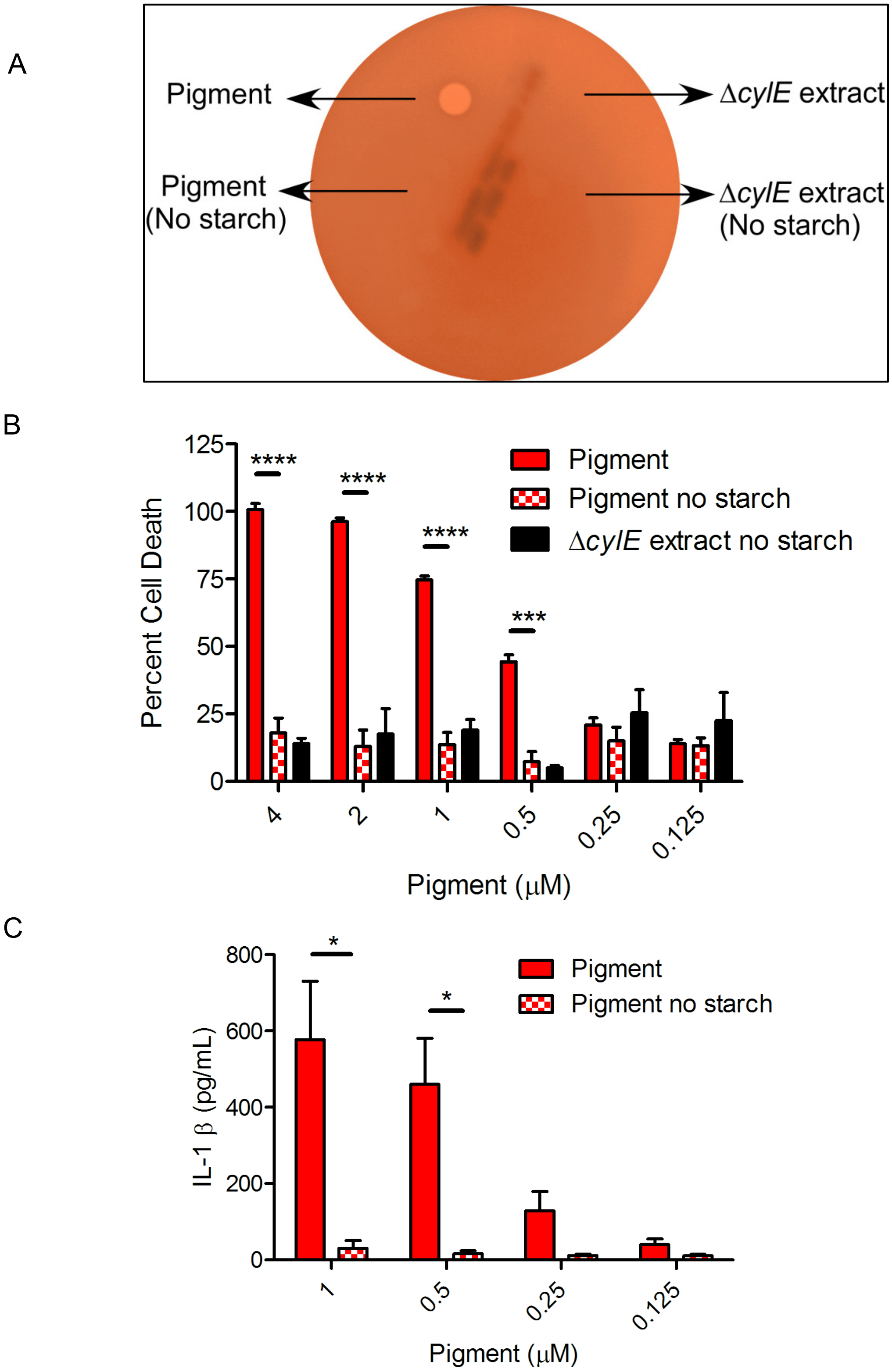

Reference Cited

Lembo A, Gurney MA, Burnside K, Banerjee A, de los Reyes M, Connelly JE, Lin WJ, Jewell KA, Vo A, Renken CW, Doran KS, Rajagopal L (2010) Regulation of CovR expression in Group B Streptococcus impacts blood-brain barrier penetration. Mol Microbiol 77**:** 431-443
